# CRISPR/Cas12a-Based Aptasensor for Cas9 Protein Detection in Spiked Food Matrices

**DOI:** 10.3390/molecules31203607

**Published:** 2026-10-10

**Authors:** Zizhao Wang, Jing Wang, Ling Li, Guofeng Chen, Hongtao Wen, Baohai Liu, Yiyang Dong

**Affiliations:** 1 College of Life Science and Technology, Beijing University of Chemical Technology, Chaoyang District, Beijing 100029, China; 2025211050@buct.edu.cn; 2 Quality and Safety Institute of Agricultural Products, Heilongjiang Academy of Agricultural Sciences/Key Laboratory of Quality and Safety of Cereals and Their Products, Harbin 150086, China; buyijingjing@163.com (J.W.); hljjiance@163.com (G.C.); wen0891@163.com (H.W.); 3 Hangzhou Institute of Medicine (HIM), The Chinese Academy of Sciences, Hangzhou 310022, China; 17864305336@163.com

**Keywords:** Cas9 protein, CRISPR/Cas technology, aptamer

## Abstract

Residual gene editors are a concern associated with the widespread application of CRISPR/Cas9 technology. Here, we developed a CRISPR/Cas12a-based fluorescent aptasensor for the qualitative and quantitative detection of Cas9 protein. Secondary structure prediction and molecular docking were combined to identify potential binding sites of the Cas9-specific aptamer. A fluorescence aptasensor was constructed based on competitive binding: in the presence of Cas9, the aptamer binds to the target and releases complementary DNA (cDNA), which activates the trans-cleavage activity of Cas12a. Cas12a subsequently cleaves a single-stranded DNA (ssDNA) reporter labeled with a fluorophore and a quencher, generating a fluorescence signal, expressed in relative fluorescence units (RFU), that increases with Cas9 concentration. The optimized conditions comprised a trans-cleavage reaction time of 20 min, a Cas12a:CRISPR RNA (crRNA) molar ratio of 1:1, a final aptamer concentration of 180 nM, and a combination of low- and high-salt buffers to balance molecular recognition and protein stability. The proposed assay enabled Cas9 protein detection in buffer and Cas9-spiked soybean milk and cereal matrices. These results represent a proof-of-concept evaluation in complex food matrices rather than validation of authentic residual Cas9 detection. Further studies using unspiked gene-edited samples with independently verified Cas9 residues and an orthogonal reference method are required.

## 1. Introduction

A genetically modified organism (GMO) is an organism whose genetic composition has been artificially altered, including through the introduction of exogenous genes, in ways that generally do not occur through natural mating or recombination. GMOs include plants, animals, and microorganisms and are widely used in agriculture, medicine, and environmental management [1]. For example, introducing a barley chitinase gene and an Aspergillus antifungal protein gene into wheat can reduce the incidence of powdery mildew and the formation of leaf rust spores. Gene gun-mediated co-transformation has also been used to generate genetically stable transgenic wheat resistant to wheat yellow mosaic disease [2]. Researchers at the Institute of Crop Science, Chinese Academy of Agricultural Sciences, used CRISPR/Cas9 to knock out SBEIIA in the winter wheat cultivar ‘Zhengmai 7698’ and the spring wheat cultivar ‘Bobwhite’, generating germplasm with modified starch properties and increased resistance. These findings provide an approach to developing functional wheat varieties [3]. A team led by Jiankang Zhu at the Shanghai Institute of Plant Stress Biology, Chinese Academy of Sciences, edited rice PYL genes encoding abscisic acid (ABA) receptors using CRISPR/Cas9, thereby increasing rice yield [4]. A team led by Caixia Gao at the Institute of Genetics and Developmental Biology, Chinese Academy of Sciences, used TALEN and CRISPR/Cas9 technologies to introduce targeted mutations into wheat MLO genes and obtain wheat with broad-spectrum resistance to powdery mildew. The team also achieved site-specific gene insertion in wheat, with stable inheritance of the inserted fragments. These advances provide approaches to investigating wheat gene function and developing new varieties and germplasm [5].

Gene editing enables precise targeted modification of specific DNA sequences within an organism’s genome. Engineered tools are used to delete, insert, or replace DNA sequences, thereby altering genetic information and biological traits. Compared with conventional transgenic approaches, gene editing allows targeted changes to the genome and can be performed without introducing exogenous genetic material [6].

The main gene editing approaches include zinc finger nucleases (ZFNs) [7], transcription activator-like effector nucleases (TALENs), clustered regularly interspaced short palindromic repeats/CRISPR-associated protein (CRISPR/Cas) systems [8], base editing, and prime editing. ZFNs combine engineered protein-DNA recognition with nuclease dimerization to introduce targeted double-strand breaks (DSBs) at specific genomic loci. TALENs use programmable TALE protein arrays to bind to specific DNA sequences and induce DSBs through FokI nuclease dimerization, activating cellular DNA repair pathways to introduce genetic modifications. CRISPR/Cas, whose development was recognized by the 2020 Nobel Prize in Chemistry [9], has accelerated the transition from conventional transgenesis to targeted genome editing. In the CRISPR/Cas9 system, a guide RNA (gRNA) directs the Cas9 nuclease to a specific DNA sequence. Cas9 induces a DSB, which is repaired through non-homologous end joining (NHEJ) or homology-directed repair (HDR), enabling gene knockout or insertion. Its simplicity and low cost have made CRISPR/Cas9 a widely used editing tool. Base editors [10], including cytosine base editors (CBEs) and adenine base editors (ABEs), enable C-to-T and A-to-G substitutions, respectively, without generating DSBs. This reduces insertion/deletion (indel) byproducts and facilitates the construction of disease models involving point mutations. Prime editing [11] combines a Cas9 nickase with a reverse transcriptase and uses a prime editing guide RNA (pegRNA) to direct base substitutions and small insertions or deletions without requiring DSBs. Its precision and relatively low off-target activity make it a promising genome editing strategy.

A major risk associated with gene editing is off-target activity, in which editing components recognize and cleave non-target DNA, causing unintended genetic modifications. Such activity may lead to mutations, deletions, rearrangements, or oncogene activation; immune responses are an additional concern associated with editing components [12]. Another concern is the persistence of gene editors after the intended editing has been completed. Persistent editing components can occur as DNA, RNA, or protein. DNA persistence may involve the integration of plasmid or viral vector fragments into the genome; RNA persistence may involve gRNA or mRNA used to express editing components; and protein persistence may involve Cas9, base editors, or other editing proteins. These residues may present several risks. Continued expression or activity of residual editors may cause unintended editing at non-target sites, potentially disrupting gene function or contributing to tumorigenesis or developmental abnormalities [13]. Editing components derived from bacteria may also elicit host immune responses, affecting treatment safety [14]. In germline or germplasm editing, persistent editing components may be transmitted to subsequent generations through gametes, raising ethical and ecological concerns. Residue detection is also challenging because standardized procedures are lacking.

Given the widespread use of CRISPR/Cas9, this study focused on developing a proof-of-concept method for Cas9 protein detection.

CRISPR/Cas systems exhibit diverse functions depending on the Cas proteins involved. They are broadly divided into two classes according to the organization of their effector complexes. Class 1 systems use multiprotein effector complexes, whereas Class 2 systems use a single effector protein. Class 2 systems are widely used and extensively studied. The Cas9 and Cas12 proteins used in this study belong to Class 2 systems. Both recognize and cleave target nucleic acids, although their mechanisms differ.

CRISPR/Cas9 is widely used for genome editing, whereas CRISPR/Cas12a has been extensively applied in molecular detection. For example, Cas12a-based assays can detect major viral pathogens in apple trees within a few hours [15]. Cas12a has also been combined with specific crRNAs and fluorescent reporters to detect meat adulteration [16]. Other CRISPR/Cas12a-based assays have been developed to detect influenza A virus, influenza B virus, and SARS-CoV-2, providing approaches to pathogen screening [17]. An assay combining reverse transcription-enzymatic recombinase amplification (RT-ERA) with CRISPR/Cas12a targets the N gene of respiratory syncytial virus (RSV) and detects both RSV A and RSV B. Amplification and detection are performed at a constant temperature within 35 min, without requiring complex instrumentation or specialized operators [18]. However, conventional CRISPR/Cas12a assays primarily target nucleic acids and do not directly recognize non-nucleic-acid analytes [19]. Extending these assays to other analytes therefore requires a recognition element that converts target binding into a nucleic acid signal. Aptamers can fulfill this role.

Aptamers are DNA or RNA oligonucleotides isolated from libraries by systematic evolution of ligands by exponential enrichment (SELEX) and can bind specifically to their targets [20]. Target binding involves intermolecular interactions and may induce conformational changes in the aptamer, supporting selective molecular recognition [21]. Integrating aptamers with CRISPR/Cas12a therefore provides a versatile sensing platform for a range of analytes [19]. For example, an aptamer-mediated CRISPR/Cas12a biosensing system has been developed for the rapid detection of the small-molecule marine toxin okadaic acid (OA). This approach provides a basis for extending CRISPR/Cas12a-based detection to other non-nucleic-acid targets.

Previous studies have established several approaches for Cas9 protein detection. Xu et al. used Western blotting and an automated capillary-based nano-immunoassay to monitor Cas9 persistence in genome-edited cells [22]. These methods provide complementary analytical capabilities, with Western blotting offering molecular-weight information alongside immunoreactivity and automated capillary immunoassays facilitating instrument-assisted protein analysis. Phaneuf et al. developed a portable centrifugal microfluidic platform that combined chemiluminescent protein detection with a fluorescence-based nuclease activity assay, enabling the detection of Cas9 orthologs from several bacterial species and achieving a detection limit of 2 pg/mL for Streptococcus pyogenes Cas9 [23]. These advances provide a foundation for exploring alternative recognition and signal-transduction strategies for Cas9 detection. In this study, we integrated a Cas9-specific aptamer with CRISPR/Cas12a to detect Cas9 protein and preliminarily evaluate the assay in Cas9-spiked food matrices.

## 2. Materials and Methods

### 2.1. Materials and Instruments

All DNA oligonucleotides were synthesized by Sangon Biotech (Shanghai, China). The Cas9-specific aptamer sequence was CGTAGAATTCATGAGGACGTTGGTGCGCCGTGTTTCCGAACAGCTAATATCATAGCCCCACAGCTAAGCTTACCAGTGCGA [24]. The corresponding CRISPR RNA (crRNA), UAAUUUCUACUAAGUGUAGAUCAUGAGGACGUUGGUGCGCC, was also synthesized by Sangon Biotech. The complementary DNA (cDNA) sequence was GGCGCACCAACGTCCTCATG. The Cas9 protein standard was purchased from New England Biolabs (Ipswich, MA, USA). The crRNA consisted of a 21-nucleotide scaffold region at its 5′ end (5′-UAAUUUCUACUAAGUGUAGAU-3′), which mediates Cas12a binding, followed by a 20-nucleotide spacer region (5′-CAUGAGGACGUUGGUGCGCC-3′). The spacer was designed to be fully complementary to the entire cDNA sequence (5′-GGCGCACCAACGTCCTCATG-3′). The cDNA was complementary to nucleotides 10–29 of the Cas9 aptamer, a region predicted by molecular docking to participate in Cas9 binding. Because the crRNA spacer corresponds to the aptamer sequence at positions 10–29, with uracil replacing thymine, it is not complementary to the aptamer itself but is complementary to the released cDNA.

Fluorescence measurements were performed using a SpectraMax M2 microplate reader supplied by Beijing Wuzhou Dongfang Technology Development Co., Ltd. (Beijing, China).

### 2.2. Computational Prediction of Potential Cas9–Aptamer Interaction Regions

Potential binding sites were investigated by combining secondary structure prediction with molecular docking [21]. The three-dimensional structure of Cas9 was obtained using AlphaFold3, whereas the initial three-dimensional model of the aptamer was generated using RNAComposer. The Cas9–aptamer complex was first predicted using AlphaFold. Molecular docking was subsequently performed using MOE to further investigate possible binding poses and interaction residues. The structures were prepared and optimized before docking, and the results obtained using AlphaFold and MOE were compared to identify consistently predicted interaction regions. These computational analyses were used to predict potential Cas9–aptamer binding sites rather than to experimentally validate them.

### 2.3. Trans-Cleavage Assay for Cas9 Detection Using the Aptasensor

The fluorescent reporter (5′-FAM-TTTTTT-BHQ1-3′) was labeled with the fluorophore FAM at the 5′ end and the quencher BHQ1 at the 3′ end. The Cas9 aptamer and cDNA were mixed at a molar ratio of 1:1 and incubated at room temperature for 10 min. Cas9 protein at different concentrations was then added to the aptamer/cDNA mixture, followed by incubation for a further 10 min. In parallel, Cas12a and crRNA were mixed at a molar ratio of 1:1 and incubated at 37 °C for 30 min to form the Cas12a/crRNA complex. Following aptamer-Cas9 binding, the Cas12a/crRNA complex was added to the reaction mixture, and a second incubation was performed. The assay was conducted in a microplate, and the reaction mixtures were protected from light.

### 2.4. Data Collection and Processing

Fluorescence intensity increased linearly with target concentration within the investigated range. Under optimized conditions, emission spectra were recorded from 510 to 650 nm at an excitation wavelength of 494 nm, with the maximum emission observed at 520 nm. Fluorescence intensity was used as the analytical response for the quantitative detection of Cas9. The assay was straightforward to perform and provided an interpretable signal for target quantification.

### 2.5. Data Processing and Quality Control

Fluorescence measurements were evaluated within individual runs and across independent runs. Measurements were excluded only when an identifiable technical problem was documented, such as incomplete reagent addition, a recorded pipetting error, unintended light exposure, instrument interruption, or corrupted data export. No measurements, wells, or experimental runs contributing to the final analyses were excluded. All measurements obtained from the experimental runs used to generate the reported results were retained and analyzed, and no data were removed solely because they deviated from the expected experimental trend. Blank regions in the plate files represent unused wells rather than failed experiments, missing data, or excluded measurements. A reliable quantitative estimate of inter-run variability could not be calculated because the available raw files were generated under different optimization conditions and did not constitute matched independent runs performed under identical conditions. The error bars shown in the relevant figures therefore represent the mean ± SD of the three replicates defined for each corresponding experiment and should not be interpreted as a comprehensive estimate of variability across different experimental runs.

### 2.6. Seed Pretreatment and Control Experiments

In this study, gene-edited cereal seeds were used as a cereal matrix for Cas9 spike-recovery experiments, and their pretreatment procedure followed a previously reported protocol [25]. The seeds were ground at low temperature, filtered through a 0.45 μm membrane, and diluted 100-fold with 1× PBS. The resulting filtrate was stored at room temperature until analysis. Commercially purchased non-GMO soybean milk was used for spike-recovery experiments. The soybean milk was centrifuged, and the resulting supernatant was filtered through a 0.45 μm membrane, diluted 20-fold with purified water, and stored at 10 °C until analysis.

## 3. Results and Discussion

### 3.1. Working Diagram of the Fluorescent Aptasensor

**Figure 1:**
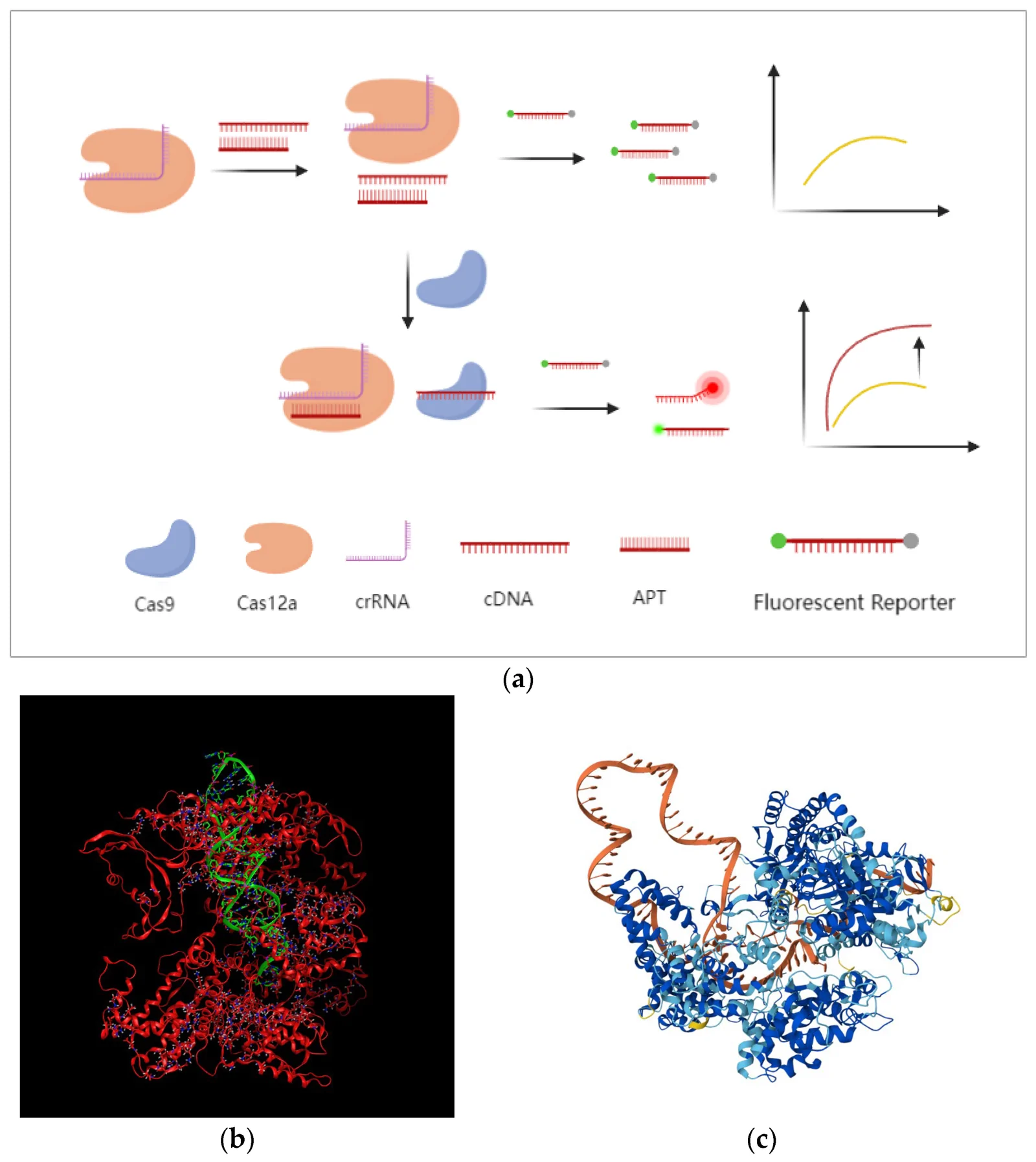
(**a**) Schematic illustration of the CRISPR/Cas12a-assisted fluorescence aptasensor (The horizontal axis represents time, and the vertical axis represents the fluorescence signal). (**b**) Molecular docking model generated using MOE (red, Cas9 protein; green, aptamer). (**c**) Molecular docking model generated using αFold (blue, Cas9 protein; orange, aptamer).

Molecular docking was used to predict the binding of the literature-reported aptamer to Cas9 and to identify potential Cas9–aptamer interaction regions, thereby providing a structural basis for the sensing strategy. Based on these docking results, aptamer nucleotides 10–29 were selected for hybridization with the complementary DNA (cDNA), such that Cas9 binding to this region would destabilize the aptamer/cDNA duplex and promote cDNA release. As illustrated in Figure 1a, the aptasensor combines competitive binding of cDNA and Cas9 to the aptamer with the trans-cleavage activity of Cas12a/crRNA. Molecular simulations were used to identify potential binding sites between the Cas9 aptamer and Cas9, guiding the design of cDNA and crRNA (Figure 1b,c). Initially, the aptamer hybridizes with cDNA through complementary base pairing. In the presence of Cas9, target binding releases cDNA into solution. The released cDNA is recognized by the Cas12a/crRNA complex, activating Cas12a-mediated trans-cleavage of the ssDNA fluorescent reporter. Cleavage separates FAM from BHQ1 and increases fluorescence intensity. Continued reporter cleavage amplifies the signal. The total reaction volume was 60 μL, comprising 20 μL of aptamer/cDNA complex prepared at a molar ratio of 1:1 (final concentration, 180 nM), 10 μL of Cas9 solution, 20 μL of the Cas12a/crRNA complex prepared at a molar ratio of 1:1 (final concentration, 50 nM), and PBS or water to make up the remaining volume. The fluorescence signal increased with Cas9 concentration. Thus, changes in fluorescence intensity enabled sensitive detection of the target. Component-omission controls showed that the fluorescence signal decreased in the absence of Cas9, cDNA, or Cas12a/crRNA, whereas free cDNA produced a strong signal in the absence of the aptamer (Figure S1). These results support the proposed Cas9-dependent cDNA-release and Cas12a trans-cleavage mechanism.

### 3.2. Optimization of Key Aptasensor Parameters

To optimize aptasensor performance, we investigated the key conditions affecting Cas12a/crRNA-mediated trans-cleavage of the fluorescent reporter. All experiments were performed in triplicate.

**Figure 2:**
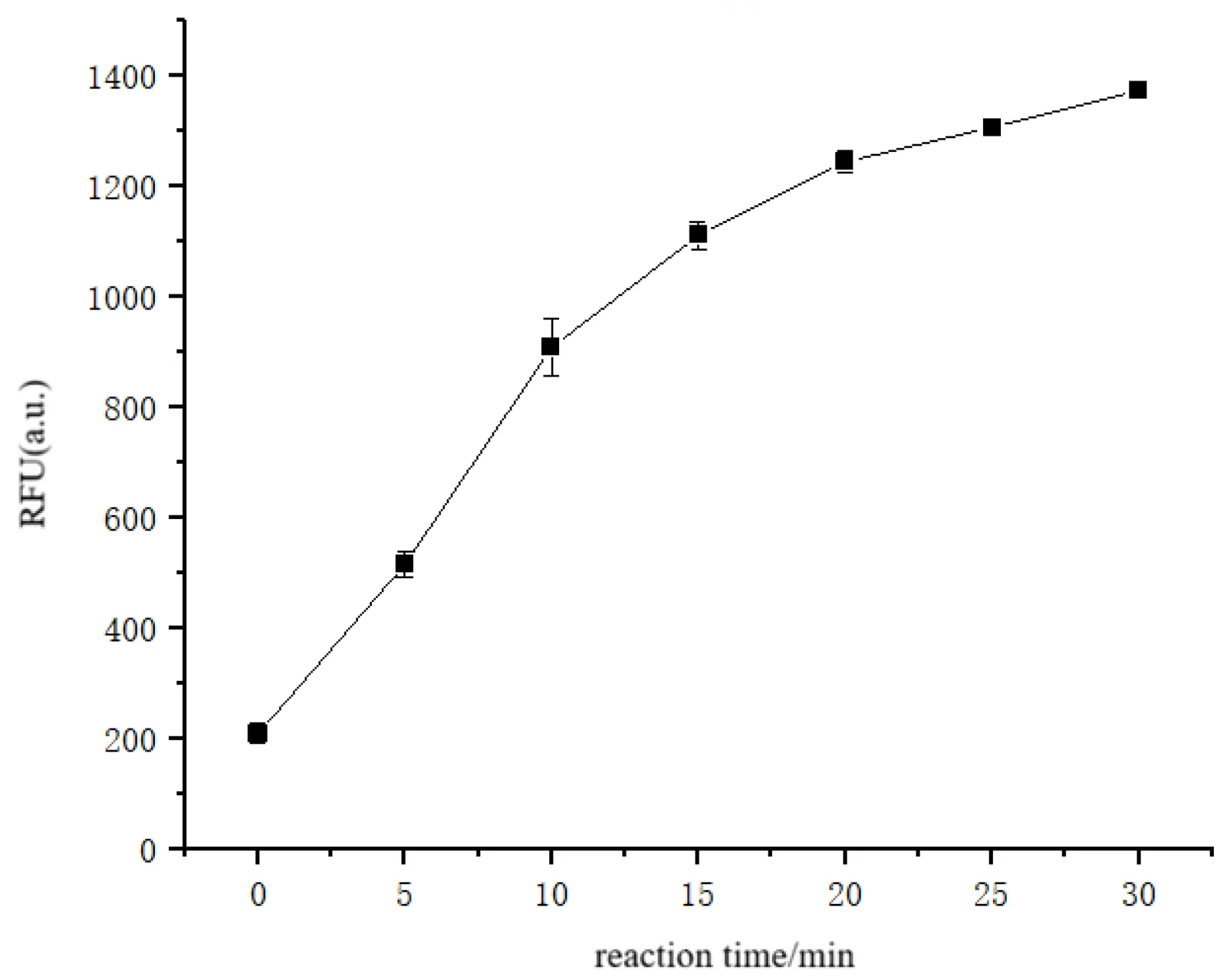
Optimization of the trans-cleavage reaction time.

Reaction time is an important determinant of assay speed. As shown in Figure 2, fluorescence intensity increased with reaction time and approached a plateau at 15–20 min. This trend may reflect depletion of the fluorescent reporter as cleavage proceeds, which reduces the rate of subsequent cleavage. A reaction time of 20 min was therefore selected to balance signal generation and assay duration.

**Figure 3:**
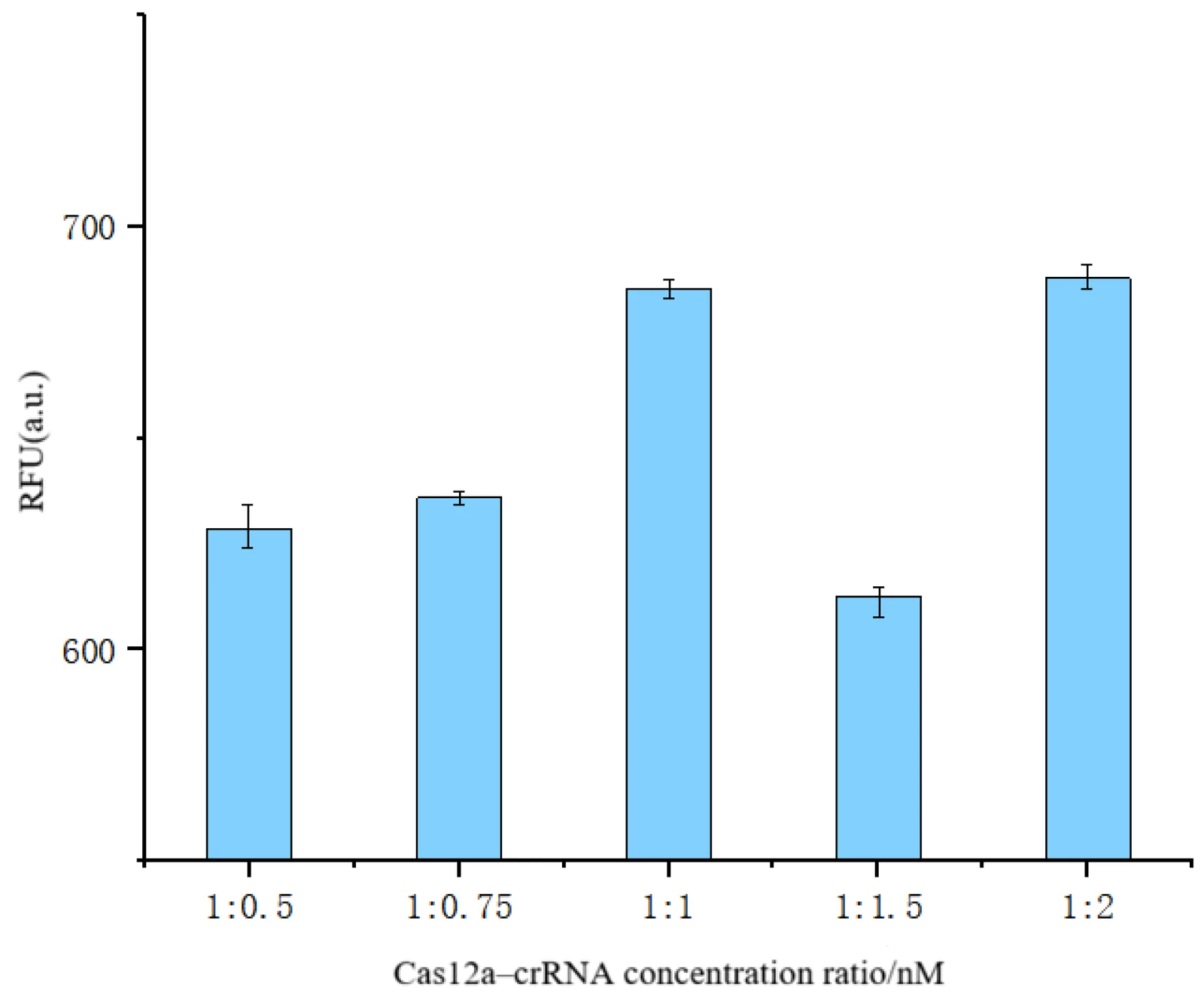
Optimization of the Cas12a:crRNA molar ratio.

The molar ratio of Cas12a to crRNA is another important assay parameter. crRNA directs target recognition, whereas Cas12a cleaves the fluorescent reporter after activation. The Cas12a/crRNA binary complex recognizes complementary DNA and initiates trans-cleavage. Its concentration therefore influences the amount of active Cas12a and the resulting fluorescence signal. Optimal Cas12a:crRNA ratios vary among CRISPR-based detection systems; the ratios reported for HOLMES and DETECTR are 1:2 and 4:5. In this study, Cas12a:crRNA molar ratios of 1:0.5, 1:0.75, 1:1, 1:1.5, and 1:2 were evaluated. As shown in Figure 3, optimal responses were obtained at Cas12a:crRNA ratios of 1:1 and 1:2. Considering reagent cost, a ratio of 1:1 was selected for subsequent experiments.

**Figure 4:**
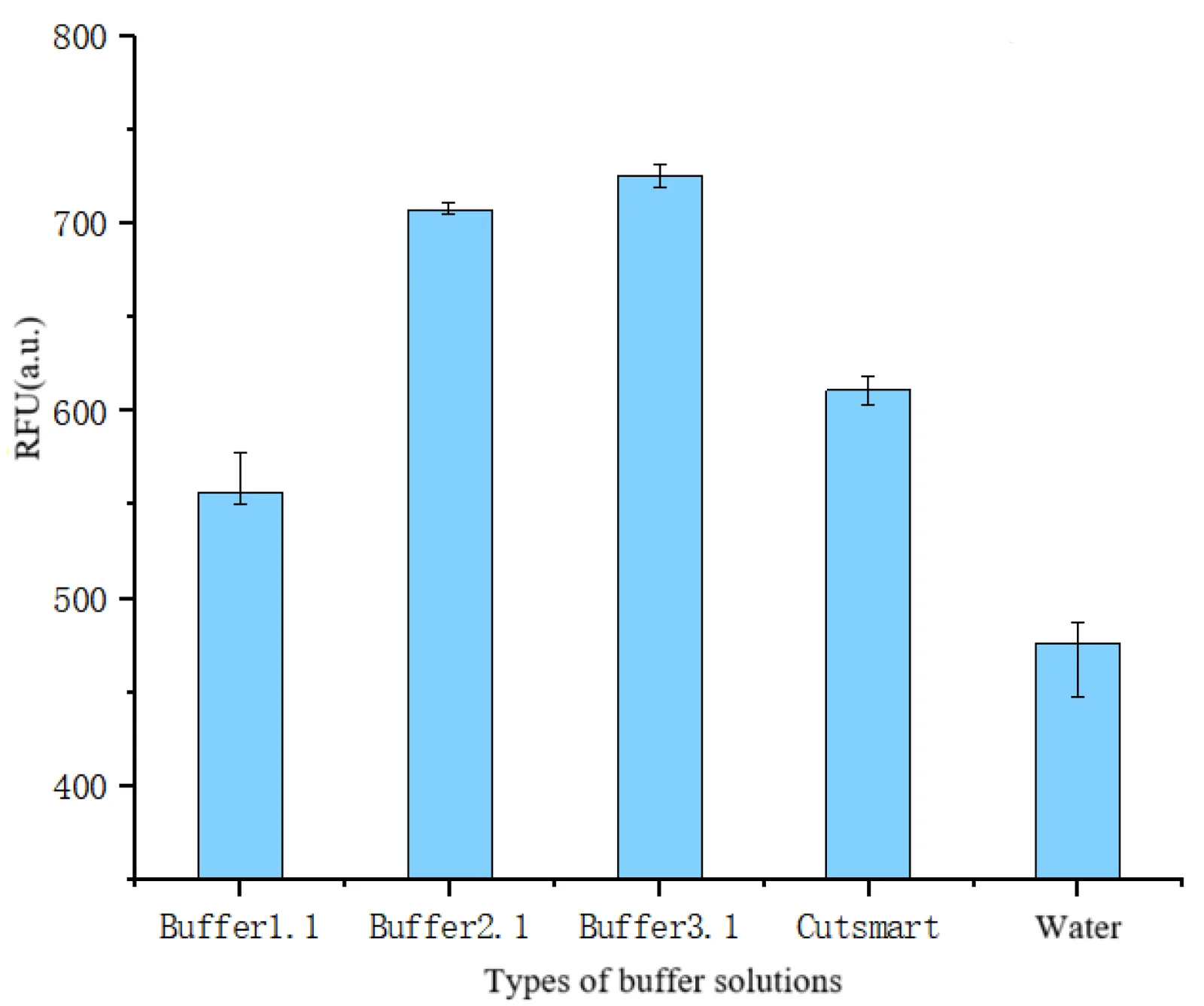
Optimization of the reaction buffer.

Buffer composition also affects Cas12a/crRNA complex formation and cleavage activity. We evaluated four reaction buffers (Buffer 1.1, Buffer 2.1, Buffer 3.1, and CutSmart), with water as a control. As shown in Figure 4, all four buffers enhanced Cas12a activity relative to water, and Buffer 2.1 and Buffer 3.1 produced comparable responses. These two buffers had similar components but different salt concentrations: Buffer 2.1 provided a low-salt environment, whereas Buffer 3.1 provided a high-salt environment. The low-salt buffer supported Cas12a/crRNA complex formation, whereas the high-salt buffer helped stabilize the proteins after Cas9 addition. Accordingly, Buffer 2.1 was used for Cas12a/crRNA assembly, followed by the addition of an equal proportion of Buffer 3.1 for protein stabilization.

**Figure 5:**
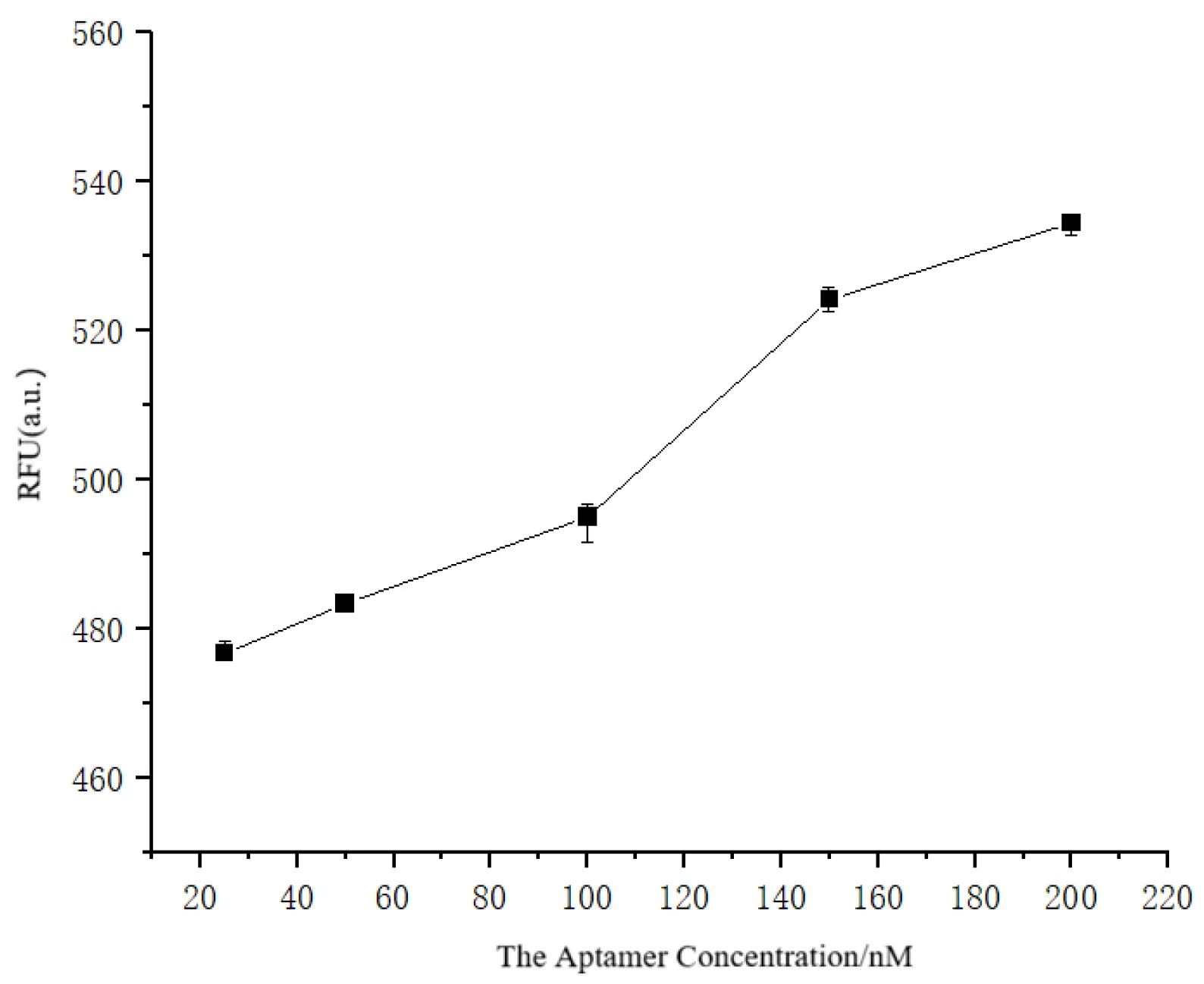
Optimization of the aptamer concentration.

The aptamer/cDNA concentration was also optimized because it affects activation of the Cas12a/crRNA complex. To support efficient trans-cleavage, the aptamer/cDNA concentration was maintained above that of the Cas12a/crRNA complex. With the Cas12a/crRNA concentration fixed at 50 nM, the response shown in Figure 5 began to plateau at an aptamer/cDNA concentration of 150 nM. A final concentration of 180 nM, within the investigated range of 150–200 nM, was selected for subsequent experiments.

### 3.3. Calibration Curve and Limit of Detection

**Figure 6:**
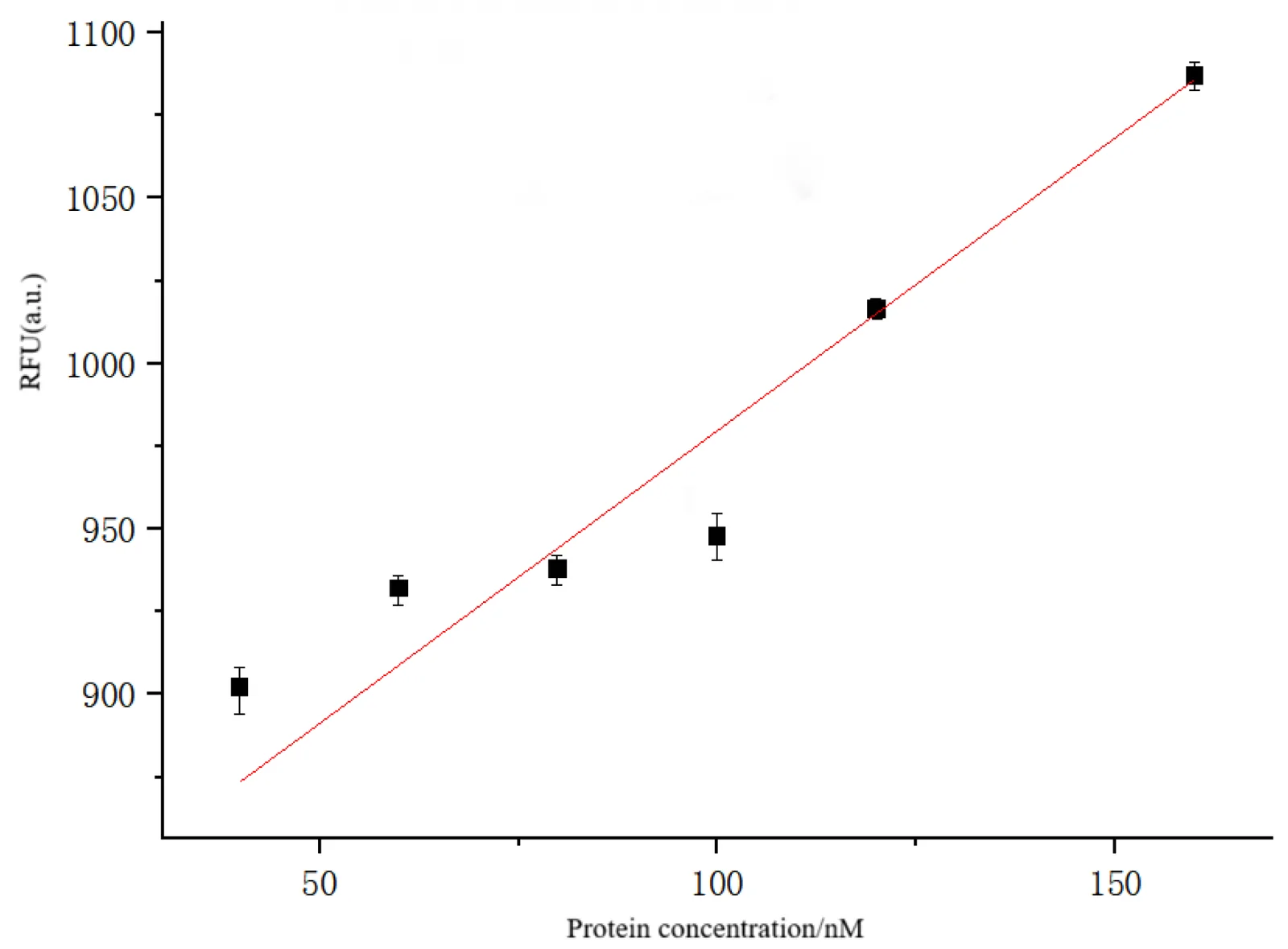
Calibration curve for Cas9 detection.

Under optimized conditions, the fluorescence response of the aptasensor was evaluated using Cas9 solutions ranging from 40 to 160 nM. As shown in Figure 6, fluorescence intensity at 520 nm increased with Cas9 concentration. The fluorescence spectra are presented in Figure 6. Fluorescence intensity was linearly correlated with Cas9 concentration over the range of 40–160 nM. The calibration equation was as follows:

*Y* = 1.7715*X* + 802.61. (1)

The coefficient of determination (R^2^) was 0.96249. The limit of detection (LOD) was calculated according to

*LOD* = 3*σ*/*S*, (2)

where σ represents the standard deviation of the fluorescence responses obtained from independent blank measurements (*n* = 10) and S represents the slope of the calibration curve. Based on an experimentally determined σ of 17.12 RFU, the LOD was calculated to be 28.99 nM, indicating the sensitivity of the aptasensor for Cas9 detection. Based on the concentration relationship of the Cas9 stock solution used in this study (20,000 nM corresponding to 3.22 mg/mL), this molar concentration was equivalent to a Cas9 concentration of approximately 0.00466 mg/mL (4.66 μg/mL) in the assay solution. No comparison with a regulatory residue limit was made because an internationally recognized numerical limit specifically applicable to residual Cas9 protein in gene-edited cereal products could not be identified.

These results indicate that the fluorescence aptasensor provides a straightforward method for sensitive Cas9 protein detection.

### 3.4. Spike-Recovery and Selectivity Evaluation

To evaluate assay performance in a complex food matrix, soybean milk samples were spiked with Cas9 at the indicated concentrations, and spike-recovery experiments were performed. As shown in Table 1, recoveries ranged from 98.106% to 104.84%, with relative standard deviations (RSDs) below 6%. These results support the accuracy and reproducibility of Cas9 detection in Cas9-spiked soybean milk samples.

**Figure 7:**
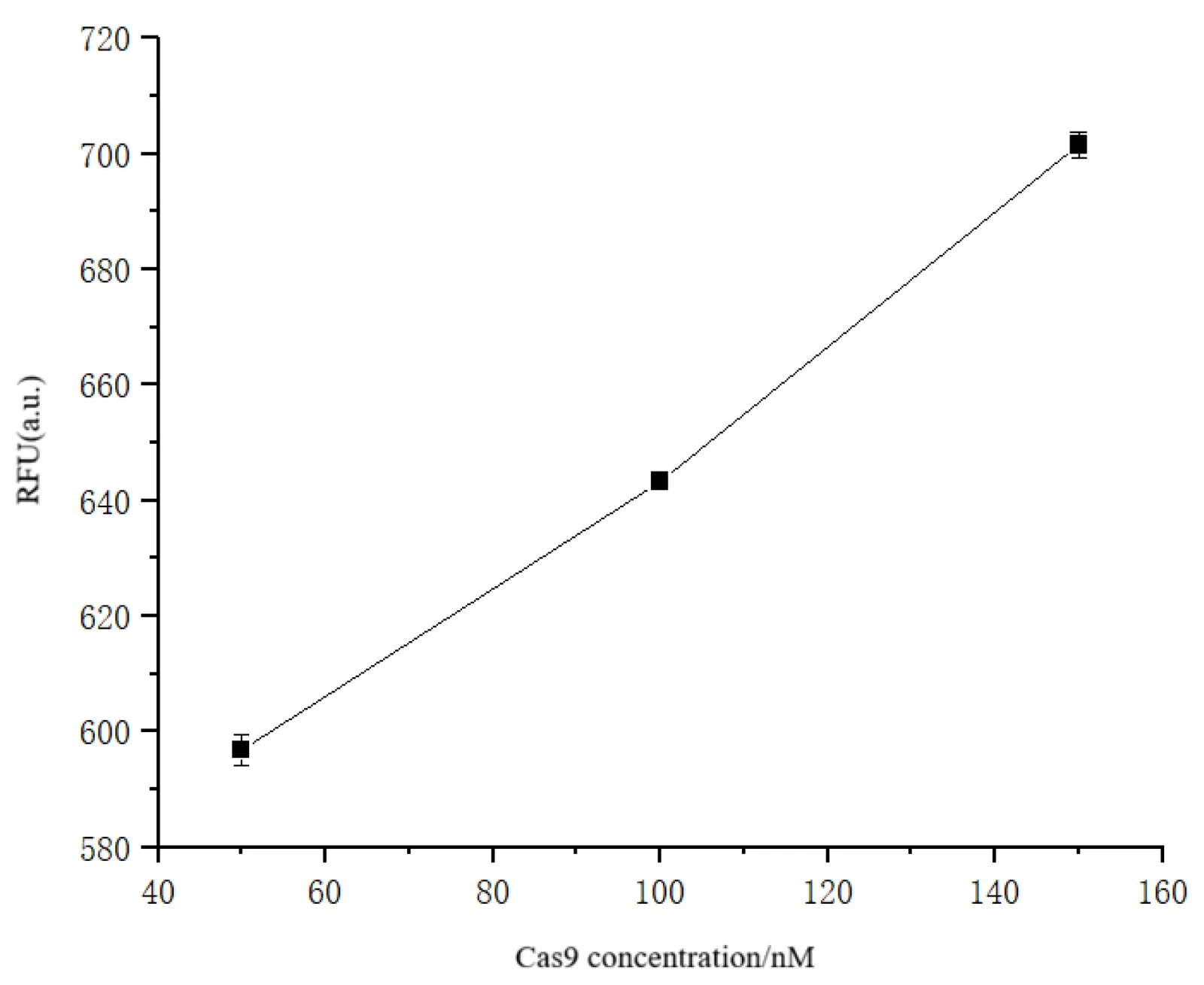
Fluorescence responses obtained from Cas9-spiked cereal matrix samples.

To preliminarily evaluate the performance of the aptasensor in a cereal matrix, gene-edited cereal seed samples were divided into three aliquots and spiked with Cas9 at final concentrations of 50, 100, and 150 nM, respectively. The resulting fluorescence responses are shown in Figure 7. The fluorescence intensities obtained from the Cas9-spiked cereal samples were lower than those obtained from the spiked soybean milk samples but retained an approximately concentration-dependent response. The reduced signals may be attributable to matrix interference from the cereal extracts. These findings demonstrate the detection of exogenously added Cas9 in a cereal matrix but do not establish the presence or concentration of authentic Cas9 residues resulting from the genome-editing process.

**Figure 8:**
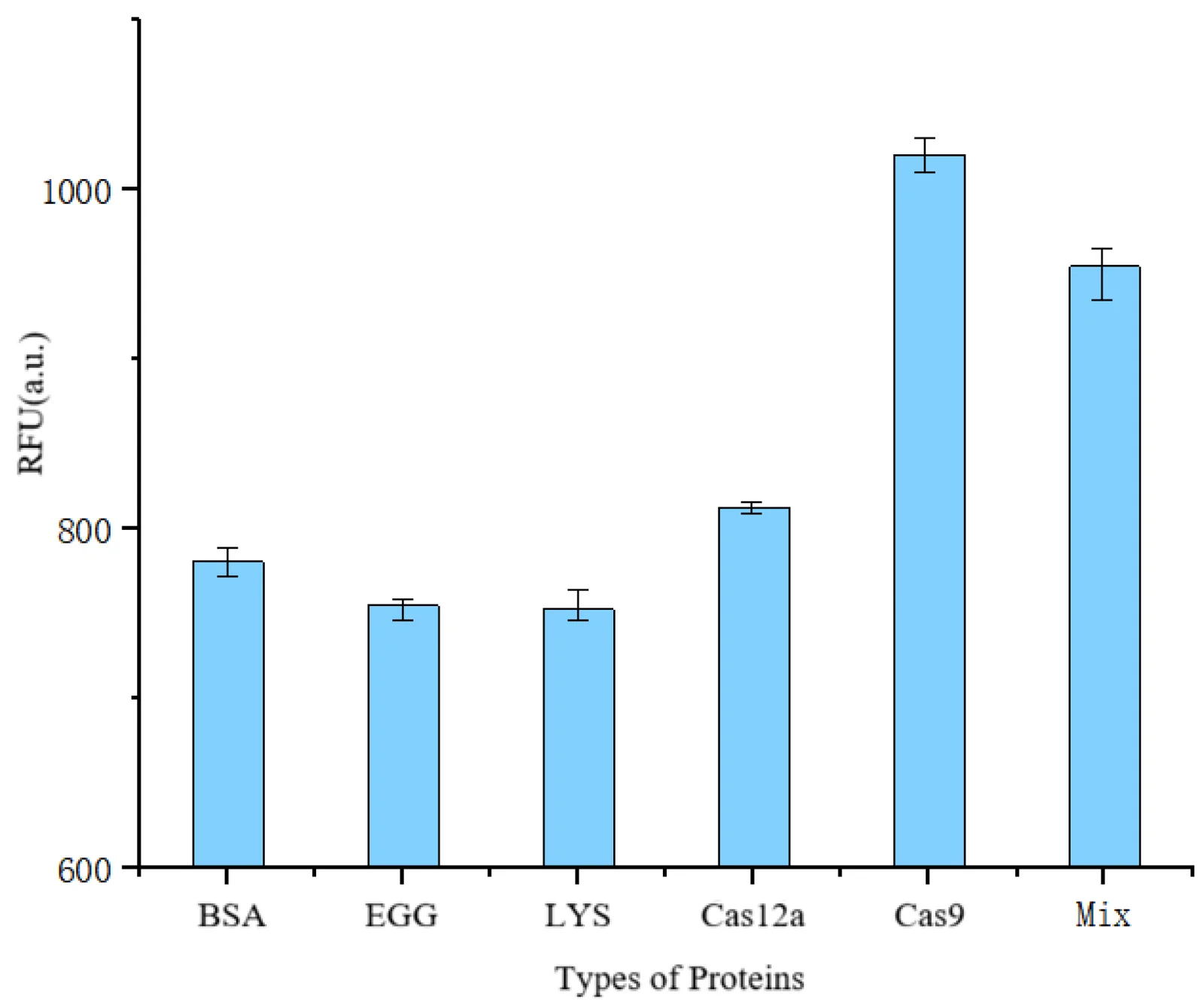
Selectivity of the developed aptasensor.

To evaluate aptasensor selectivity, potentially interfering food-associated proteins, including bovine serum albumin (BSA), ovalbumin (EGG), lysozyme (LYS), and Cas12a, were analyzed at the same concentration (100 nM). As shown in Figure 8, the apparent Cas9 concentration measured for Cas9-containing samples was substantially higher than that measured for the other proteins, tested individually or as a mixture of four proteins. These results suggest that the aptasensor selectively recognizes Cas9 in the presence of potential interferents.

## 4. Conclusions

In this study, we developed an aptamer-mediated CRISPR/Cas12a fluorescence assay for Cas9 protein detection. The assay combines aptamer–target recognition with Cas12a-mediated trans-cleavage and was evaluated in buffer and Cas9-spiked food matrices. Under the optimized conditions, the assay exhibited a linear response over 40–160 nM, with an LOD of 28.99 nM. The spike-recovery results obtained from Cas9-spiked soybean milk samples provide preliminary evidence that the assay can operate in a complex food matrix.

The optimized assay conditions comprised a trans-cleavage reaction time of 20 min, a Cas12a:crRNA molar ratio of 1:1, a final aptamer concentration of 180 nM, and a combination of low-salt Buffer 2.1 and high-salt Buffer 3.1. These conditions balanced Cas12a/crRNA complex assembly with Cas9 protein stability. The assay exhibited a linear response over the range of 40–160 nM, with a coefficient of determination (R^2^) of 0.96249 and an LOD of 28.99 nM. Recoveries in Cas9-spiked samples ranged from 98.106% to 104.84%, with RSDs below 6%. The assay produced approximately concentration-dependent responses in Cas9-spiked gene-edited cereal seed samples, although the fluorescence intensities were lower than those obtained in spiked soybean milk samples, potentially because of matrix interference. These results provide preliminary evidence that the assay can detect exogenously added Cas9 in cereal matrices. The assay also showed selectivity for Cas9 in the presence of interfering proteins, including BSA, ovalbumin, and lysozyme.

The proposed assay provides a proof-of-concept approach for Cas9 protein detection in buffer and Cas9-spiked food matrices. Its sensitivity and selectivity may support further development of assays for product quality and safety assessment. Additional validation across sample types and relevant regulatory contexts would help establish its suitability for routine monitoring by regulatory agencies and biotechnology companies. However, the present study did not establish the presence or quantify the concentration of authentic residual Cas9 in unspiked gene-edited cereal samples. Further validation using unspiked samples with independently verified Cas9 residues and an orthogonal reference method is required before the assay can be applied to authentic residue monitoring.

## Figures and Tables

**Table 1:** Spike recovery of Cas9 in Cas9-spiked soybean milk samples (*n* = 3).

Sample	Theoretical Concentration (nM)	Theoretical Fluorescence Intensity (RFU a.u.)	Mean Measured Fluorescence Intensity (RFU a.u.)	Calculated Concentration (nM)	Recovery	RSD
1	50	891.185	874.306	49.053	98.106%	5.38%
2	100	979.76	984.85	100.52	100.52%	2.12%
3	150	1068.335	1120.046	157.26	104.84%	5.578%

## Data Availability

All data can be found at https://doi.org/10.6084/m9.figshare.33328818.

## Supplementary Materials

The following supporting information can be downloaded at: https://www.mdpi.com/article/10.3390/molecules31203607/s1: original data. Figure S1: RFU of various solution components for feasibility confirmation.
